# Aqueous Molecular Dynamics Simulations of the *M. tuberculosis* Enoyl-ACP Reductase-NADH System and Its Complex with a Substrate Mimic or Diphenyl Ethers Inhibitors

**DOI:** 10.3390/ijms161023695

**Published:** 2015-10-07

**Authors:** Camilo Henrique da Silva Lima, Ricardo Bicca de Alencastro, Carlos Roland Kaiser, Marcus Vinícius Nora de Souza, Carlos Rangel Rodrigues, Magaly Girão Albuquerque

**Affiliations:** 1 Graduate Program in Chemistry, Institute of Chemistry (Instituto de Química), Federal University of Rio de Janeiro (Universidade Federal do Rio de Janeiro, UFRJ), 21949-900 Rio de Janeiro, RJ, Brazil; E-Mails: bicca@iq.ufrj.br (R.B.A.); kaiser@iq.ufrj.br (C.R.K.); marcos_souza@far.fiocruz.br (M.V.N.S.); 2 Oswaldo Cruz Foundation (Fundação Osvaldo Cruz, FioCruz), Institute of Pharmaceutical Technology (Instituto de Tecnologia em Fármacos, FarManguinhos), 21041-250 Rio de Janeiro, RJ, Brazil; 3 Graduate Program in Pharmaceutical Sciences, College of Pharmacy (Faculdade de Farmácia), Federal University of Rio de Janeiro, 21949-900 Rio de Janeiro, RJ, Brazil; E-Mail: rangel@pharma.ufrj.br

**Keywords:** *Mycobacterium tuberculosis*, enoyl-ACP reductase (InhA), molecular dynamics simulation, diphenyl ethers inhibitors, triclosan derivatives, water-bridge hydrogen bond

## Abstract

Molecular dynamics (MD) simulations of 12 aqueous systems of the NADH-dependent enoyl-ACP reductase from *Mycobacterium tuberculosis* (InhA) were carried out for up to 20–40 ns using the GROMACS 4.5 package. Simulations of the holoenzyme, holoenzyme-substrate, and 10 holoenzyme-inhibitor complexes were conducted in order to gain more insight about the secondary structure motifs of the InhA substrate-binding pocket. We monitored the lifetime of the main intermolecular interactions: hydrogen bonds and hydrophobic contacts. Our MD simulations demonstrate the importance of evaluating the conformational changes that occur close to the active site of the enzyme-cofactor complex before and after binding of the ligand and the influence of the water molecules. Moreover, the protein-inhibitor total steric (E_LJ_) and electrostatic (E_C_) interaction energies, related to Gly96 and Tyr158, are able to explain 80% of the biological response variance according to the best linear equation, p*K*_i_ = 7.772 − 0.1885 × **Gly96** + 0.0517 × **Tyr158** (*R*^2^ = 0.80; *n* = 10), where interactions with Gly96, mainly electrostatic, increase the biological response, while those with Tyr158 decrease. These results will help to understand the structure-activity relationships and to design new and more potent anti-TB drugs.

## 1. Introduction

Tuberculosis (TB) is an infecto-contagious disease caused by *Mycobacterium tuberculosis* (MTB), mainly affecting lungs, but it can also infect others vital organs, such as central nervous, genitourinary, and osteoarticular systems [1,2,3]. In 2013, according to the World Health Organization (WHO), about 1.5 million people die every year from TB, and this disease is the second cause of death worldwide [4]. Since the 1990s, the WHO recommends the DOTS (directly observed treatment, short-course) strategy that includes a chemotherapy regimen combining four first-line drugs (*i.e*., isoniazid, rifampicin, ethambutol, and pyrazinamide) for at least six to nine months [5,6]. Despite this scheme having high rates of a successful cure, many patients discontinue treatment due to serious side effects, such as hepatotoxicity, which can be fatal [4,7].

The main consequence of the TB treatment abandonment is the emergence of multi-drug resistant (MDR) strains, showing resistance to at least isoniazid and rifampicin, and extensively-drug resistant (XDR) strains, with the same profile of MDR strains plus resistance to a fluoroquinolone (e.g., ofloxacin or ciprofloxacin) and at least one injectable drug (e.g., amikacin, kanamycin or capreomycin) [8,9]. As a result, chemotherapy required against MDR-TB and XDR-TB has several disadvantages, e.g., it is less effective, more expensive, and more toxic in comparison to the first-line drugs [9].

**Figure 1:**
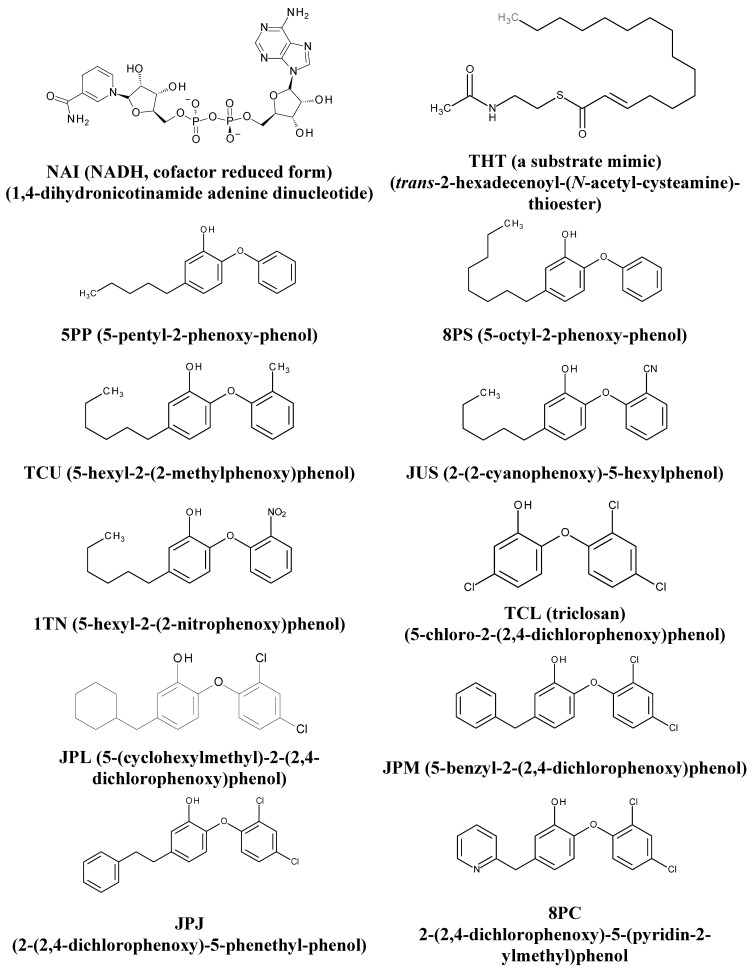
2D chemical structures of NAI (NADH, cofactor reduced form), THT (a substrate mimic), and diphenyl ethers inhibitors.

Thus, due to the prolonged treatment, toxicity, and emergence of resistant strains to the anti-TB drugs, it is necessary to develop new, more potent and effective drugs. Therefore, the enoyl-acyl carrier protein (ACP) reductase (ENR) is an attractive target to design new drugs, because it is an essential enzyme of the type II fatty acid synthesis (FAS-II) [10]. The ENR from *M. tuberculosis* (InhA) catalyzes the reduction of *trans*-2-enoyl-ACPs that is dependent of the NADH cofactor (Figure 1). In fact, the anti-tuberculosis drug isoniazid acts as a pro-drug, activated by oxidation catalyzed by *M. tuberculosis* catalase-peroxidase (KatG). Hence, this product and cofactor (NADH or NAD+) react to form an adduct that inhibits InhA, disrupting the biosynthesis of mycolic acids (FAS-II), the main components of the mycobacterial cell wall, thus causing cell death [11].

The required activation of isoniazid is responsible for the emergence of isoniazid-resistant strains related to KatG mutations [12,13,14]. This fact led to efforts to identify direct InhA inhibitors that do not require activation through KatG [15,16,17,18]. In this context, diphenyl ethers are a promising class because they can directly inhibit InhA. Triclosan (5-chloro-2-(2,4-dichlorophenoxy)phenol, TCL (Figure 1), an important representative of this class, is an antimicrobial (antibacterial and antifungal) agent found in toothpaste and deodorants, which inhibits ENRs in pathogenic organisms, such as *Escherichia coli* and *Staphylococcus aureus* [19,20,21].

Recently, 3D structures of several diphenyl ethers inhibitors, including TCL and TCU (Figure 1 and Table 1), in ternary complexes with InhA and the oxidized cofactor form (NAD+), solved by X-ray diffraction and available in the Protein Data Bank (PDB; http://www.rcsb.org/pdb/) [22], allowed researchers to describe the main H-bonding and hydrophobic enzyme-inhibitor interactions in the substrate binding pocket [23,24]. Importantly, these data come from crystallization experiments using only the oxidized form of the cofactor, where the enzyme-cofactor-inhibitor ratio is 1:5:200 [25].

**Table 1:** Description of the InhA-cofactor-ligand complexes used as starting structures in the molecular dynamics simulations.

PDB ID ^a^	R (Å) ^b^	Cofactor ^c^	Ligand ^c^	p*K*_i_ (M) ^d^	Ref.
2AQ8	1.92	NAI	n.d	n.d	[26]
1BVR	2.80	NAD	THT	n.d	[27]
2X23	1.81	NAD	TCU	9.86	[25]
2B37	2.60	NAD	8PS	8.47	[23]
3FNF	2.30	NAD	JPM	7.93	[24]
2B36	2.80	NAD	5PP	7.91	[23]
3FNE	1.98	NAD	8PC	7.78	[24]
3FNH	2.80	NAD	JPJ	7.52	[24]
4OXY	2.35	NAD	1TN	7.22	[28]
3FNG	1.97	NAD	JPL	7.17	[24]
4OIM	1.85	NAD	JUS	6.77	[16]
2B35	2.30	NAD	TCL	6.16	[23]

^a^ The Protein Data Bank (PDB) identification code. ^b^ Resolution (Angstrom). ^c^ The PDB ligand identification codes: cofactor, NAI=NADH or NAD=NAD+; a substrate mimic, THT; and diphenyl ethers inhibitors (TCU, 8PS, JPM, 5PP, 8PC, JPJ, 1TN, JPL, JUS, and TCL). ^d^ The original half-maximal inhibitory concentration (IC_50_) was converted to the inhibition constant (K_i_) [29], which was converted to p*K*_i_ (*i.e.*, –Log*K*_i_). n.d = not determined.

In the IC_50_ assays, fixed concentrations of InhA, the substrate mimic (DD-CoA, Figure 1), and reduced cofactor are used to evaluate the conversion rate from NADH to NAD+ [25]. For example, TCL shows IC_50_ = 1000 nM [23], but this inhibitor has a greater affinity for the enzyme-NAD+ complex with NAD+ pre-incubation, according to kinetic studies with ENR from *E. coli* and *B. napus* (FabI) [30], and *M. tuberculosis* (InhA) [31]. TCU shows IC_50_ = 5.3 and 50.3 nM at InhA concentrations equal to 10 and 100 nM, respectively [25], in a kinetic study considering only the oxidized cofactor step.

New derivatives of this class have been synthesized and evaluated [16], but none showed IC_50_ better than that reported for TCU. It is noteworthy that these studies aim to find compounds which dissociate slowly from the InhA-NAD+ complex generated after catalysis [16,25,28]. This proposed inhibition mechanism is related to α-helix-6 motion, in the substrate-binding pocket, via a slow conformational conversion from closed to open states. However, in these studies, it was not possible to determine clearly the influence of structural changes in the diaryl ethers class with this motion. Furthermore, some authors argue that maintaining the α-helix-6 structure is directly related with the inhibitor residence time in the InhA-cofactor-inhibitor complex, influencing the biological response [23,25,28,32,33,34]. Recent isothermal titration calorimetry and thermal melting studies with other classes of InhA inhibitors, such as methyl-thiazol [35] and NITD-564 [36], have shown the importance in evaluating whether inhibition occurs with the enzyme apo (free InhA) or holoenzyme (InhA-NADH or InhA-NAD+) forms. Both inhibitors, methyl-thiazol (IC_50_ = 3 nM) [35] and NITD-564 (IC_50_ = 590 nM), bind preferentially to InhA-NADH [36].

Moreover, homologous enzymes to InhA, such as FabI from *E. coli*, *B. natus*, and *P. falciparum*, show marked differences [10,25,26,37,38,39]: (i) FabI accepts substrates with carbon chain length from 4 to 18 carbons, while InhA prefers substrates with 12–24 carbons. (ii) FabI accepts both unphosphorylated and phosphorylated cofactors, while InhA recognizes NADH, but not NADPH. (iii) Different substrate mimics are used in kinetic studies for FabI (crotonyl-CoA, 4 carbons) and InhA (DD-CoA, 12 carbons). Thus, results from kinetic studies with InhA homologous enzymes should be carefully interpreted.

The hypothesis that diphenyl ethers bind to the enzyme-NAD+ complex generated after catalysis, which is consistent with kinetic data from *in vitro* assays, does not rule out the hypothesis that, *in vivo*, the inhibitor binds to the enzyme-NADH complex before catalysis, since there is a gap between *in vitro* assays and the actual biological environment. Therefore, in the current work, molecular dynamics (MD) simulations in aqueous solvent of the holoenzyme (InhA-cofactor), holoenzyme-substrate, and 10 holoenzyme-inhibitor systems were performed considering the cofactor reduced form, in order to gain more insight about the solvent influence on the H-bond and hydrophobic interactions, and the dynamic behavior of the secondary structures that compose the binding site. The results obtained from this MD study could help to design new, more potent and effective InhA inhibitors in order to improve the pharmacological treatment against TB.

## 2. Results and Discussion

In order to evaluate the main structural changes that lead to the inhibition of the NADH-dependent enoyl-ACP reductase enzyme from *Mycobacterium tuberculosis* (InhA), we carried out up to 20 or 40 ns of molecular dynamics (MD) simulations of 12 aqueous protein systems, using the GROMACS 4.5 package, by investigating one binary (holoenzyme) and 11 ternary (holoenzyme-ligand) complexes. The ligands are THT (a substrate mimic) and 10 potent InhA inhibitors from the diphenyl ether class, including TCL (triclosan), which were divided into two groups: TCL derivatives (TCL, JPL, JPM, JPJ, and 8PC) and alkyl diphenyl ethers (5PP, 8PS, TCU, JUS, and 1TN). The starting point for the MD simulations are the corresponding X-ray crystal structures of the binary (InhA-cofactor) and ternary (InhA-cofactor-ligand) complexes available in the Protein Data Bank (PDB). The holoenzyme (InhA-NADH) and holoenzyme-substrate (InhA-NADH-THT) complexes were used as reference for the evaluation of the holoenzyme-inhibitor complexes. The MD study was conducted in order to gain more insight into the enzyme dynamic behavior before and after binding to the ligand (substrate or inhibitor), focusing on the secondary structure motifs close to the substrate binding pocket of the InhA active site. Therefore, in order to understand the structure-activity relationships (SAR) into the design of new and more potent anti-TB drugs, it was monitored the lifetime of the main intermolecular interactions in these systems: hydrogen bonds (including water-bridge H-bonds) and hydrophobic contacts between protein and ligands.

### 2.1. Comparative Analysis of the MD Simulations of the Holoenzyme (InhA-NADH) and Holoenzyme-Substrate (InhA-NADH-THT) Complexes

**Figure 2:**
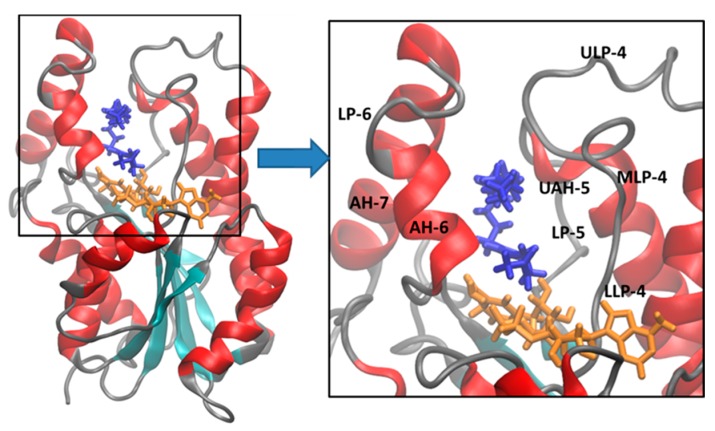
Cartoon of the 3D structure of the NADH-dependent enoyl-ACP reductase from *Mycobacterium tuberculosis* (InhA) in complex with the cofactor (blue) and a substrate mimic (THT, orange) (PDB ID: 1BVR), and a close view (box) of the substrate binding pocket, showing the following secondary structure domains: LLP-4 (Ser94-Pro99), MLP-4 (Gln100-Ile105), ULP-4 (Asn106-Asp110), LP-5 (Phe149-Ala157), UAH-5 (Tyr158-Lys165), AH-6 (Leu197-Val203), LP-6 (Gly204-Gly208), and AH-7 (Glu209-Arg225). Protein secondary structure color code: red (α-helix), cyan (β-sheet), and grey (loop or irregular or coil).

In order to simplify the analysis of the substrate binding pocket motion within the enzyme active site during the MD simulations, this pocket was divided in eight domains: lower loop-4 (LLP-4, Ser94-Pro99), middle loop-4 (MLP-4, Gln100-Ile105), upper loop-4 (ULP-4, Asn106-Asp110), loop-5 (LP-5, Phe149-Ala157), upper α-helix-5 (UAH-5, Tyr158-Lys165), α-helix-6 (AH-6, Leu197-Val203), loop-6 (LP-6, Gly204-Gly208), and α-helix-7 (AH-7, Glu209-Arg225). Figure 2 shows these domains using the InhA-cofactor-THT X-ray crystal structure (PDB ID: 1BVR).

**Figure 3:**
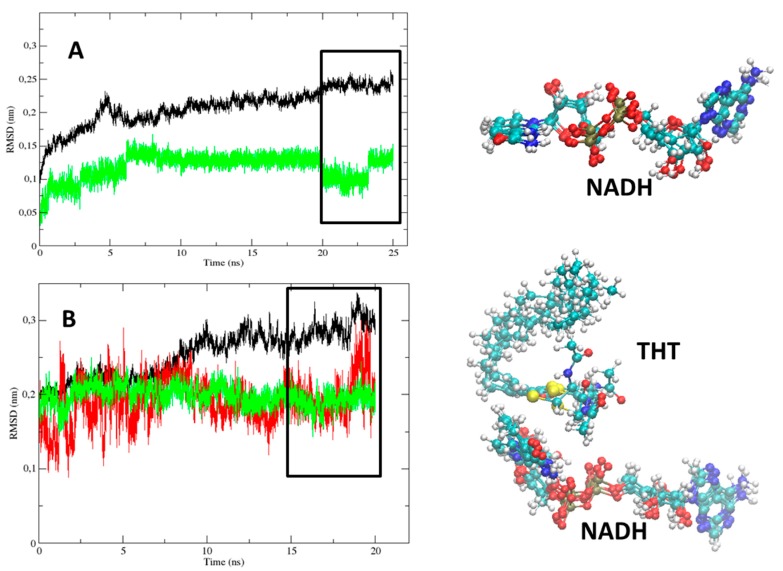
The root-mean-square deviation (RMSD, nm) of InhA protein (Cα-atoms, black line), NADH cofactor (non-hydrogen atoms, green line), and THT substrate mimic (non-hydrogen atoms, red line) during the MD simulations (Time, ns). The (**A**) InhA-NADH (2AQ8) and (**B**) InhA-NADH-THT (1BVR) complexes, and on the right, the corresponding NADH and THT conformations of the last 5 ns of each simulation. Atoms color code: carbon = cyan, nitrogen = blue, oxygen = red, hydrogen = white, phosphorus = tan, and sulfur = yellow.

The structural stability of both protein complexes, 2AQ8 (InhA-NADH) and 1BVR (InhA-NADH-THT), was monitored (Figure 3). In 2AQ8 (Figure 3A), the InhA (black line) and cofactor (green line) atoms fluctuations tend to reach a plateau before 10 ns, but only after 20 ns of simulation the protein and NADH RMSD values reached the established criteria (rectangle). Analyzing the conformational change of NADH in the last 5 ns of simulation, the main variations occur in the pentose and phosphate groups. In 1BVR (Figure 3B), the protein reach a plateau at approximately 10 ns of simulation; however, according to the established criteria, the RMSD fluctuation reached a value of less than 1.5 Å faster than 2AQ8. Analyzing the last 5 ns, the cofactor heavy atoms showed RMSD values below 0.5 Å, with the major variations occurring in the nicotinamide group located near the substrate mimic. Regarding THT, the average RMSD is 1.6 Å, but the irregularities shown in Figure 3B are due to large conformational freedom of the linear alkyl group, since the alkene group has little deviation of atomic positions in the last 5 ns of simulation.

**Figure 4:**
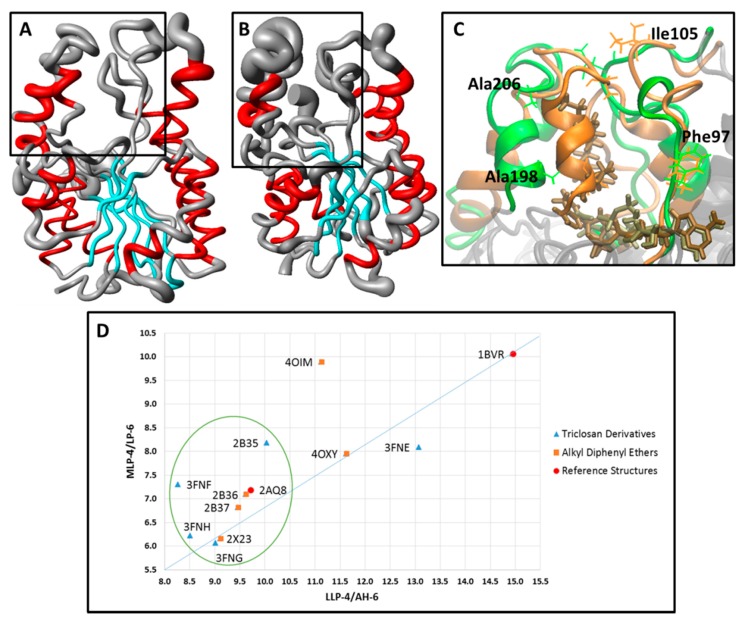
Spatial RMSD (RMSF) of (**A**) InhA-NADH (2AQ8) and (**B**) InhA-NADH-THT (1BVR) (protein secondary structure color code: α-helix, red; β-sheet, cyan; loop, grey) during the MD simulations. (**C**) Close view of the substrate binding pocket overlap between InhA-NADH (orange) and InhA-NADH-THT (green), showing the Phe97 (LLP-4), Ala198 (AH-6), Ile105 (MLP-4), and Ala206 (LP-6) residues used for measure the LLP-4/AH-6 and MLP-4/LP-6 distances to characterize the protein open and closed states. (**D**) Graphic of the MLP-4/LP-6 *versus* LLP-4/AH-6 distances. The points within the circle represent complexes where the enzyme adopts a closed conformation as in 2AQ8.

In order to obtain more structural details about the dynamic behavior of the protein, the local structure flexibility was monitored by means of the spatial RMSD (*i.e*., the RMSF of the α-C atoms throughout the last 5 ns of simulation), aiming to evaluate the local atoms fluctuation mainly close to the substrate binding pocket (Figure 4). Therefore, the spatial RMSD of 2AQ8 and 1BVR are depicted as a tubular backbone model, where less flexible regions (low RMSD values) are represented by thinner tubes, while more flexible regions (high RMSD values) are represented by wider tubes.

The analysis of the spatial RMSD of 2AQ8 (Figure 4A) shows little fluctuation of residues belonging to the substrate binding pocket. However, AH-6 suffered partial denaturation changing to a 3_10_-helix conformation (Leu197-Ser200), while AH-7 suffered partial unfolding (Glu209-Ile215). In 1BVR (Figure 4B), THT seems to increase the mobility of LP-6 and AH-7, mainly comprising Val203 to Gly221, without changing the secondary structure of AH-6. Increased mobility in these two domains may be related to large fluctuation of the linear alkyl group of THT due to its high conformational freedom. Interestingly, studies with EcFabI-NADH-TCL (PDB ID: 1QSG) [40] and FtuFabI-NAD-TCL (PDB ID: 3NRC) [41] show a type of lid, above the nicotinamide ring, which prevents access to the solvent. In the case of InhA, this lid would correspond to the intersection region of LP-6 and AH-7.

In order to characterize different conformational states (e.g., open and closed) [33,34], related to a lid protecting access to the substrate binding pocket, interatomic distances between the pairs of residues Phe97/Ala198 (LLP-4/AH-6) and Ile105/Ala206 (MLP-4/LP-6) were measured in the average structures of 2AQ8 and 1BVR, which are shown superimposed in Figure 4C. The LLP-4/HA-6 and MLP-4/LP-6 distances (Figure 4D and Table 2) are smaller in InhA-NADH (9.71 and 7.19 Å, respectively) than in InhA-NADH-THT (14.95 and 10.06 Å, respectively). These data indicate that InhA adopts a closed conformational state in the InhA-NADH complex, where a lip protects the substrate-binding pocket (Figure 4A), and an open conformational state in the InhA-NADH-THT complex, where the substrate is exposed to the solvent due to the large distance between MLP-4 and LP-6. Together, these results point to the importance of evaluating conformational changes [42,43,44] and structural disorder [45,46] of the enzyme in order to understand the binding affinity and dissociation of the ligand.

**Table 2:** LLP-4/AH-6 and MLP-4/LP-6 interatomic distances evaluated in all complexes to characterize the open and closed states of the InhA protein.

# ^a^	LLP-4/AH-6 ^b^	MLP-4/LP-6 ^c^	# ^a^	LLP-4/AH-6 ^b^	MLP-4/LP-6 ^c^
2AQ8	9.71	7.19	1BVR	14.95	10.06
2X23	9.12	6.16	2B37	9.47	6.82
3FNF	8.27	7.30	2B36	9.63	7.09
3FNE	13.08	8.09	3FNH	8.52	6.22
4OXY	11.63	7.95	3FNG	9.02	6.06
4OIM	11.13	9.89	2B35	10.04	8.18

^a^ Complexes: 2AQ8 (InhA-NADH), 1BVR (InhA-NADH-THT), 2X23 (InhA-NADH-TCU), 2B37 (InhA-NADH-8PS), 3FNF (InhA-NADH-JPM), 2B36 (InhA-NADH-5PP), 3FNE (InhA-NADH-8PC), 3FNH (InhA-NADH-JPJ), 4OXY (InhA-NADH-1TN), 3FNG (InhA-NADH-JPL), 4OIM (InhA-NADH-JUS), 2B35 (InhA-NADH-TCL). ^b^ Interatomic distance (Å) between the alpha-carbon (α-C) atoms of Phe97 and Ala198 from LLP-4 and AH-6, respectively. ^c^ Interatomic distance (Å) between the alpha-carbon (α-C) atoms of Ile105 and Ala206 from MLP-4 and LP-6, respectively.

### 2.2. Comparative Analysis of the MD Simulations of the Holoenzyme-Inhibitor Complexes

Several experimental [16,23,25,32] and theoretical [33,34,47,48] studies of InhA in complex with inhibitors from the diphenyl ether class use as a reference an X-ray crystal structure containing the cofactor in its oxidized form (NAD+). Most of the InhA-cofactor-inhibitor complexes available in PDB were obtained by crystallization assays from an enzyme solution (saturated with an inhibitor) containing a molar excess of the cofactor in its oxidized form (NAD+) [49]. However, kinetic studies of InhA with TCL demonstrate that the interaction also occurs with NADH, and the TCL binding to InhA (wild-type) is uncompetitive with respect to both NADH and DD-CoA [50]. Therefore, all MD simulations in our work were carried out considering the cofactor in its reduced form (NADH) in order to evaluate the possibility of these diphenyl ether inhibitors bind to the InhA-NADH complex. Moreover, to prevent misinterpretations on the actual potency of these inhibitors, due to variations in the enzyme concentration on the IC_50_ assays, these values were normalized by converting to p*K*_i_ [29].

**Figure 5:**
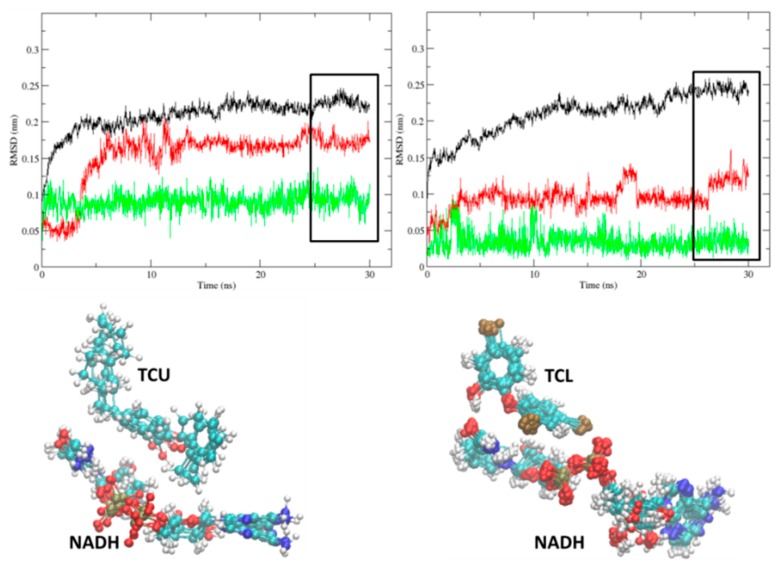
The root-mean-square deviation (RMSD, 1 Å ) of InhA protein (Cα-atoms, black line), NADH cofactor (non-hydrogen atoms, green line), and TCU and TCL inhibitors (non-hydrogen atoms, red line) during the MD simulations (Time, ns) of InhA-NADH-TCU (2X23, left) and InhA-NADH-TCL (2B35, right), and on the bottom, the corresponding NADH, TCU, and TCL conformations of the last 5 ns of each simulation.

In the temporal RMSD graphs of the holoenzyme-inhibitor complexes of the most potent (TCU, p*K*_i_ = 9.86 M) and least potent (TCL, p*K*_i_ = 6.16 M) diphenyl ethers of this series (Figure 5), NADH maintains a constant and low fluctuation level during the entire simulation time, reaching a stability plateau, in both cases, near 10 ns. In the last 5 ns of the 2X23 and 2B35 complexes simulation (rectangle, Figure 5), the protein backbone has an average RMSD equal to 1.32 Å, while TCU and TCL have an average RMSD equal to 0.85 and 0.30 Å, respectively. In InhA-NADH-TCU (2X23), the greater atomic fluctuation occurs in the alkyl group, while for InhA-NADH-TCL (2B35), the greater fluctuation corresponds to the phenol ring.

**Figure 6:**
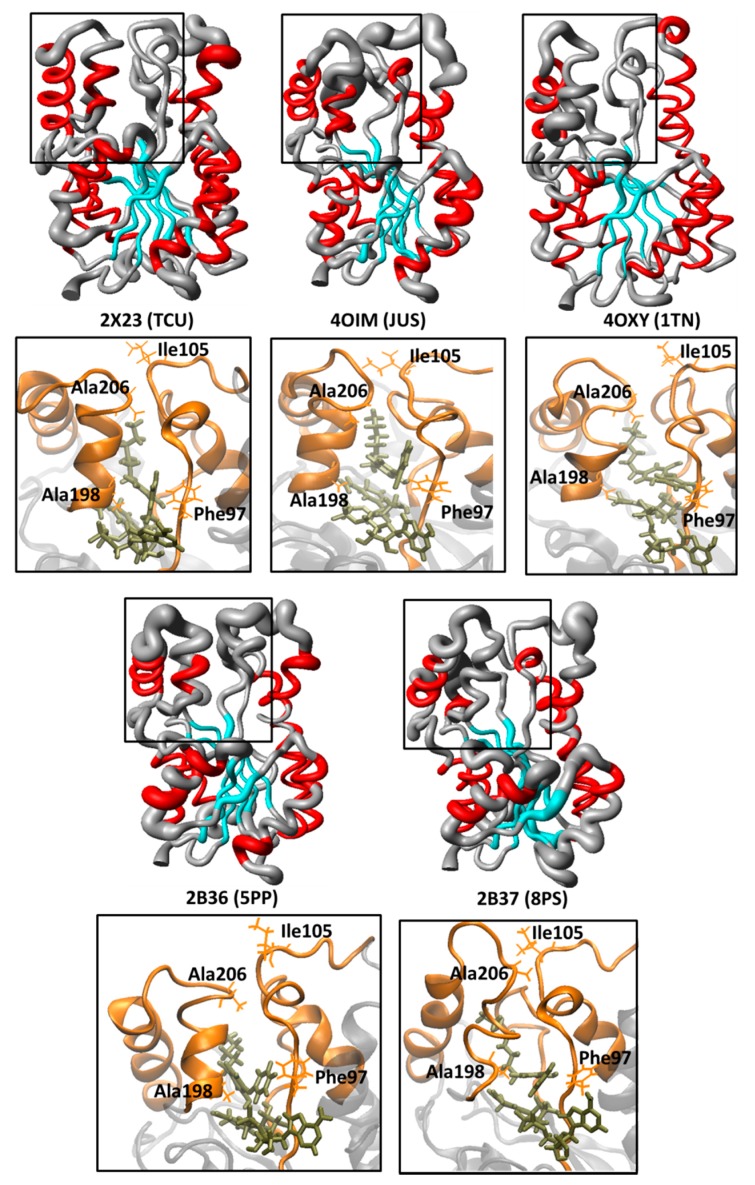
Spatial RMSD (RMSF) of InhA-NADH (protein secondary structure color code: α-helix, red; β-sheet, cyan; loop, grey) in complex with the alkyl diphenyl ethers inhibitors (TCU, JUS, 1TN, 5PP, and 8PS) during the MD simulations and the corresponding close view of the substrate binding pocket, showing the Phe97 (LLP-4), Ala198 (AH-6), Ile105 (MLP-4), and Ala206 (LP-6) residues used for measure the LLP-4/AH-6 and MLP-4/LP-6 distances to characterize the protein open and closed states.

Figure 6 shows the spatial RMSD (RMSF) graphs of complexes 2X23 (TCU, p*K*_i_ = 9.86 M), 2B37 (8PS, p*K*_i_ = 8.47 M), 2B36 (5PP, p*K*_i_ = 7.91 M), 4OXY (1TN, p*K*_i_ = 7.22 M), and 4OIM (JUS, p*K*_i_ = 6.77 M) during the last 5 ns of simulation, considering only the alkyl diphenyl ethers subgroup (TCU, JUS, 1TN, 5PP, and 8PS), and the corresponding close view of the substrate binding pocket, showing the residues used for measure the LP-6/MLP-4 and AH-6/LLP-4 distances (Table 2) to characterize the protein open and closed states.

According to the spatial RMSD graphic of 2X23 (Figure 6), containing the most potent alkyl diphenyl ether derivative (TCU), which is also the most potent of the entire series, there is low mobility of the active site residues (rectangle), where the secondary structures of AH-6 and AH-7 are maintained. The LP-6/MLP-4 and AH-6/LLP-4 distances (Table 2, Figure 4D andFigure 6) observed in 2X23 (6.16 and 9.12 Å, respectively) are even lower than those found in 2AQ8 (InhA-NADH; 7.19 and 9.71 Å, respectively), likewise corresponding to a closed state of the enzyme. Due to spatial proximity between MLP-4 and LP-6, the inhibitor should remain protected from the solvent. However, this cavity must have some stability due to H-bond interactions between Ile105(N) and Ala206(O) with 79.7% of occupancy.

The LP-6/MLP-4 and AH-6/LLP-4 distances (Figure 4D and Table 2), which are observed in 4OIM and 4OXY (Figure 6), corresponds to an open conformation, where the lid opening (LP-6) leaves JUS and 1TN exposed to solvent, thus reducing their affinities to the holoenzyme. In 4OIM (Figure 6), the secondary structure of AH-6 is maintained from Met199 to Ile202, but there is a 3_10_-helix from Gln100 to Gly102 in MLP-4 due to H-bonds comprising Met103(N)-Gln100(O) and Gly102(N)-Pro99(O), with occupancies up to 60.6% and 96.4%, respectively. In 4OXY (Figure 6), AH-6 loses its secondary structure due to an increased movement of Leu197-Gly212, associated with the increase of the LLP-4/AH-6 distance, thus exposing the inhibitor to the solvent.

In 2B36 and 2B37, whose corresponding inhibitors (5PP and 8PS) differ only in the length of the 5-alkyl phenol chain (pentyl and octyl, respectively), the MLP-4/LP-6 and AH-6/LLP-4 distances are smaller (but close) than those found in 2AQ8 (Table 2), *i.e.*, the enzyme is in its closed conformation. Nevertheless, there is an increased mobility at AH-6 and LP-6 with partial unfolding of AH-6 (Ser200-Ala201), while in 2B37, even with the low mobility of AH-6 and LP-6, there is also a partial unfolding of AH-6 with the formation of a 3_10_-helix from Gln100 to Gly102 in the MLP-4 region (Figure 6).

**Figure 7:**
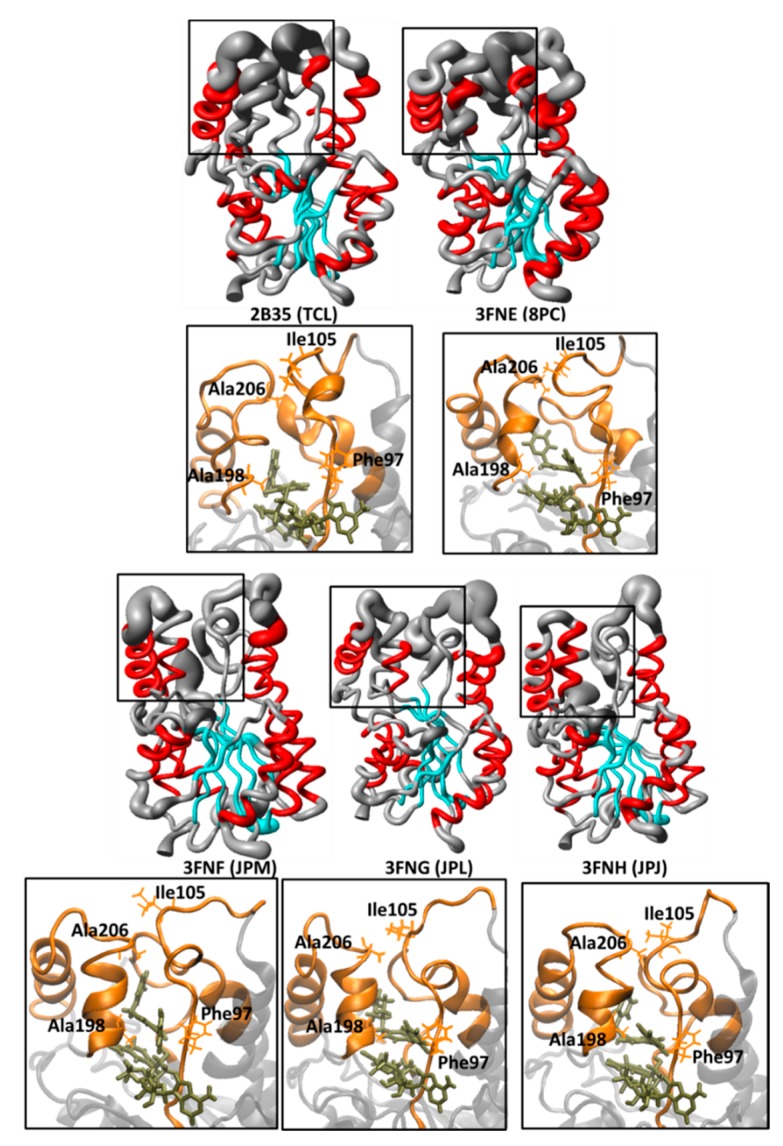
Spatial RMSD (RMSF) of InhA-NADH (protein secondary structure color code: α-helix, red; β-sheet, cyan; loop, grey) in complex with triclosan (TCL) and its derivatives (8PC, JPM, JPL, and JPJ) during the MD simulations and the corresponding close view of the substrate binding pocket, showing the Phe97 (LLP-4), Ile105 (MLP-4), Ala198 (AH-6), and Ala206 (LP-6) residues used for measure the LP-6/MLP-4 and AH-6/LLP-4 distances to characterize the protein open and closed states.

Figure 7 shows the spatial RMSD (RMSF) graphs of 2B35 (TCL, p*K*_i_ = 6.16 M), 3FNE (8PC, p*K*_i_ =7.78 M), 3FNF (JPM, p*K*_i_ = 7.93 M), 3FNG (JPL, p*K*_i_ = 7.17 M), and 3FNH (JPJ, p*K*_i_ = 7.52 M) during the MD simulations, considering only TCL and its derivatives (8PC, JPM, JPL, and JPJ), and the corresponding close view of the substrate binding pocket, showing the residues used for measure the LP-6/MLP-4 and AH-6/LLP-4 distances (Figure 4D and Table 2) to characterize the protein open and closed states.

According to the spatial RMSD graphic of 2B35 (InhA-NADH-TCL, Figure 7), containing TCL, which is the least potent compound of this subgroup (and of the entire series), there is an increased mobility of residues from ULP-4, AH-6, and LP-6, with a 3_10_-helix formation in MLP-4. In addition, there is a H-bond interaction between Gln100(NE2) and Ile202(O) from MLP-4 and AH-6, respectively, with an occupancy of 72.8%. This H-bond along with the increased mobility of LP-6 could be responsible for the overall AH-6 unfolding. Furthermore, in 2B35, the binding site adopts a closed conformation (Figure 4D) as in holoenzyme, according to AH-6/LLP-4 and LP-6/MLP-4 distances (Table 2). In fact, these results are consistent with those described for EcFabI-NADH-TCL [40] and FtuFabI-NAD-TCL [41], where the lid closing (due to the movement of AH-6) maintains the inhibitor inside the binding site.

In 3FNE (InhA-NADH-8PC) (Figure 4D and Table 2), the LP-6/MLP-4 and AH-6/LLP-4 distances indicate that the enzyme assumes an open conformation due to the high mobility of ULP-4, AH-6, and LP-6, but maintaining the secondary structure of AH-6, while MLP-4 assumes a 3_10_-helix fold (Figure 7). In 3FNF and 3FNG, whose corresponding inhibitors (JPM and JPL) differ only in the 5-position substituent of the phenol ring (benzyl and cyclohexylmethyl, respectively), ULP-4, AH-6, and LP-6 have lower mobility than in 3FNE (Figure 7). In addition, the AH-6/LLP-4 and LP-6/MLP-4 distances are even lower than those found in 2AQ8 (Figure 4D and Table 2), indicating that despite the substituent volume, the enzyme maintains a closed conformation. Finally, in 3FNH, containing JPJ (which differs from JPM and JPL by the presence of the 5-phenetyl substituent), we observed regions of low-fluctuation from the substrate binding pocket except for UPL-4 (Figure 7). In addition, the AH-6/LLP-4 and LP-6/MLP-4 distances are similar to those found in 3FNF and 3FNG (Figure 4D and Table 2), indicating again that, despite the substituent volume, the enzyme maintains a closed conformation.

### 2.3. Analysis of Holoenzima-Inhibitor Interactions by Hydrogen Bond and Hydrophobic Contact

The analysis of the H-bond and hydrophobic contact interactions occurring between the holoenzyme (InhA-NADH) and inhibitors is crucial to understanding the structural changes observed and discussed above. According to literature [23,24], Tyr158 (LP-5) is described as responsible for H-bond interaction in the substrate binding pocket, while eleven residues distributed in LP-5 (Phe149, Met155, Pro156, and Ala157), AH-5 (Met161), AH-6 (Ala198, Met199, Ile202, and Val203), and AH-7 (Leu218 and Trp222) are described as responsible for hydrophobic contacts.

**Figure 8:**
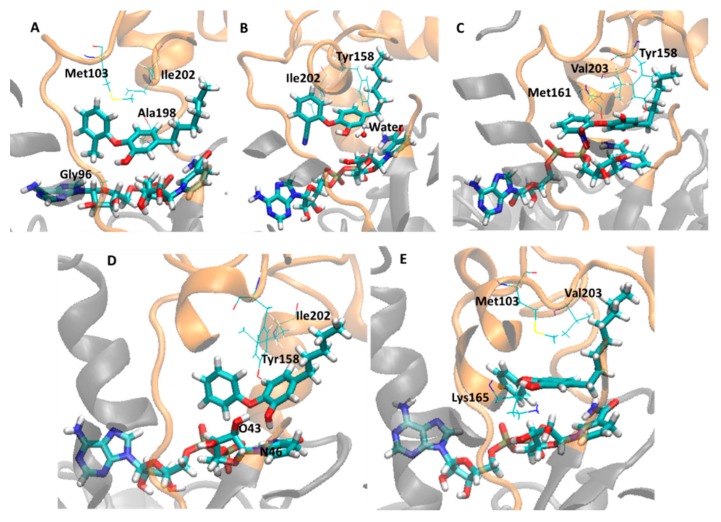
Close view of the InhA-NADH active site (orange) in complex with the alkyl diphenyl ethers inhibitors (TCU, JUS, 1TN, 5PP, and 8PS), showing important residues and water molecule. NADH and inhibitors are shown as stick models colored by element (carbon, cyan; hydrogen, white; nitrogen, blue; oxygen, red; sulfur, yellow). (**A**) InhA-NADH-TCU (2X23); (**B**) InhA-NADH-JUS (4OIM); (**C**) InhA-NADH-1TN (4OXY); (**D**) InhA-NADH-5PP (2B36); (**E**) InhA-NADH-8PS (2B37).

**Figure 9:**
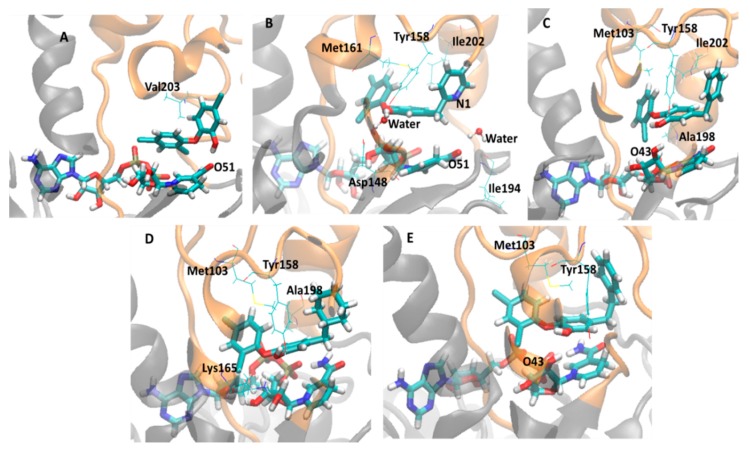
Close view of the active site of the InhA-NADH (orange) in complex with triclosan (TCL) and its derivatives (8PC, JPM, JPL, and JPJ), showing important residues and water molecule. NADH and inhibitors are shown as stick models colored by element (carbon, cyan; hydrogen, white; nitrogen, blue; oxygen, red; sulfur, yellow). (**A**) InhA-NADH-TCL (2B35); (**B**) InhA-NADH-8PC (3FNE); (**C**) InhA-NADH-JPM (3FNF); (**D**) InhA-NADH-JPL (3FNG); (**E**) InhA-NADH-JPJ (3FNH).

Thus, in order to obtain more insight about the binding mode of these inhibitors and to identify residues that actually can contribute to explain the biological activity profile of this series of compounds, it was monitored the lifetime (*i.e*., occupancy) of the main intermolecular interactions in these systems during the MD simulations, *i.e.*, hydrogen bonds (H-bonds) between holoenzyme and inhibitors, including indirect H-bonds mediated by water molecules (water-bridge), and hydrophobic contacts. Table 3 shows the monitored interactions (H-bond and hydrophobic contact) between all diphenyl ethers inhibitors and InhA-NADH during the last 5 ns of simulations. Figure 8 and Figure 9 show the close view of the substrate binding pocket with the alkyl diphenyl ethers subgroup (TCU, JUS, 1TN, 5PP, and 8PS) and TCL and its derivatives (8PC, JPM, JPL, and JPJ), respectively.

**Table 3:** Hydrogen bond and hydrophobic contact interactions between the diphenyl ethers inhibitors and InhA-NADH during the last 5 ns of the MD simulations of complexes: 2X23 (InhA-NADH-TCU), 2B37 (InhA-NADH-8PS), 3FNF (InhA-NADH-JPM), 2B36 (InhA-NADH-5PP), 3FNE (InhA-NADH-8PC), 3FNH (InhA-NADH-JPJ), 4OXY (InhA-NADH-1TN), 3FNG (InhA-NADH-JPL), 4OIM (InhA-NADH-JUS), and 2B35 (InhA-NADH-TCL).

# ^a^	Hydrogen Bond ^b^			Hydrophobic Contact ^c^		
	Donor	Acceptor	Time (%) ^d^	Residue	Distance	Time (%) ^d^
2X23 (TCU, 9.86)	OH	Gly96	26.5	Met103	3.49 (± 0.26)	92
	-	-	-	Ile202	3.80 (± 0.14)	25.6
	-	-	-	Ala198	3.83 (± 0.15)	25.2
2B37 (8PS, 8.47)	Lys165	OH	4.8	Met103	3.91 (± 0.34)	99
	-	-	-	Val203	3.57 (± 0.26)	76.7
3FNF (JPM, 7.93)	OH	Tyr158	42.0	Met103	3.73 (± 0.19)	65.7
	OH	NADH O43	27.2	Ile202	3.82 (± 0.14)	39.3
	-	-	-	Ala198	3.82 (± 0.10)	16.4
2B36 (5PP, 7.91)	NADH O43	OH	87.6	Tyr158	3.20 (± 0.25)	95.4
	OH	NADH N46	28.3	Ile202	3.88 (± 0.13)	3.4
	Tyr158	OH	5.5	-	-	-
3FNE (8PC, 7.78)	OH	HOH	98.5	Met161	3.42 (± 0.32)	92
	HOH	Tyr158	96.2	Ile202	3.74 (± 0.18)	30.5
	HOH	Asp148	76.2	-	-	-
	HOH	NADH O51	65.29	-	-	-
	HOH	N1	57.7	-	-	-
	HOH	Ile194	38	-	-	-
3FNH (JPJ, 7.52)	OH	NADH O43	25.0	Met103	2.88 (± 0.25)	100
	OH	Tyr158	23.7	-	-	-
4OXY (1TN, 7.22)	Tyr158	OH	91.4	Met161	3.70 (± 0.19)	82.4
	-	-	-	Val203	3.71 (± 0.19)	37
3FNG (JPL, 7.17)	OH	Tyr158	73.6	Met103	3.30 (± 0.32)	94.4
	Lys165	OH	67	Ala198	3.88 (± 0.10)	4.0
4OIM (JUS, 6.77)	HOH	Tyr158	98.2	Tyr158	3.72 (± 0.18)	71.1
	HOH	OH	89.2	Ile202	3.94 (± 0.07)	2.2
2B35 (TCL, 6.16)	OH	NADH O51	96.8	Val203	3.81 (± 0.14)	40.7

^a^ PDB ID of the InhA-NADH-ligand complex (ligand PDB ID, p*K*_i_, M). ^b^ The H-bonds (D-H…A) were computed considering the cutoff distance between donor (D) and acceptor (A) atoms until 0.35nm and the cutoff H-D-A angle until 30°. ^c^ The cutoff value for distance (nm) between non-hydrogen atoms of the residues and inhibitor was until 0.40 nm. ^d^ Occupancy percentage (%) considering only the last 5 ns of the MD simulation.

Considering the alkyl diphenyl ethers subgroup, in InhA-NADH-TCU (closed conformation) (2X23, Table 3, Figure 8A), there is an H-bond between the phenol hydroxyl group of TCU and the Gly96 backbone (LLP-4) with occupancy of 26.5%. In addition, this inhibitor also makes hydrophobic contacts of high and low occupancies with residues from MLP-4 (Met103, 92%) and AH-6 (Ala198, 25.2% and Ile202, 25.6%), respectively. In InhA-NADH-JUS (open conformation) (4OIM, Table 3, Figure 8B), there are H-bonds mediated by a water molecule (water-bridge H-bond) between the phenol hydroxyl group of JUS and Tyr158 (UAH-5), which are of high occupancy (HOH-inhibitor, 89.2%; HOH-Tyr158, 98.2%). This inhibitor also makes hydrophobic contacts of high and low occupancies with residues from UAH-5 (Tyr158, 71.1%) and AH-6 (Ile202, 2.2%), respectively. In InhA-NADH-1TN (open conformation) (4OXY, Table 3, Figure 8C), there is an H-bond between Tyr158 and the phenol hydroxyl group of 1TN with high occupancy (91.4%). The inhibitor also makes hydrophobic contacts of high and medium occupancies with residues from UAH-5 (Met161, 82.4%) and AH-6 (Val203, 37%), respectively. Comparing 4OXY and 4OIM, the absence of a solvent-inhibitor interaction may be related to maintaining the lid closed, even with the AH-6 motion, thus preventing solvent-inhibitor interaction.

In InhA-NADH-5PP (closed conformation) (2B36, Table 3, Figure 8D), 5PP (phenol hydroxyl group) interacts by H-bond mainly with NADH (O43, H-bond donor, 87.6%; N46, H-bond acceptor, 28.3%) and occasionally with Tyr158 (OH, H-bond donor, 5.5%). In fact, Tyr158 is mainly responsible by hydrophobic contacts with this inhibitor (95.4%), providing more stability to this complex. In addition, the inhibitor also makes minor hydrophobic contacts with Ile202 (3.4%). Finally, in InhA-NADH-8PS (closed conformation) (2B37, Table 3, Figure 8E), there is a charge-reinforced H-bond between the phenol hydroxyl group (–OH, neutral H-bond acceptor) of 8PS and the protonated amino group (–NH_3_^+^, charged H-bond donor) of Lys165 with low occupancy (4.8%), but of higher stability than neutral H-bond. The hydrophobic contacts of high occupancy with residues from MLP-4 (Met103, 99%) and AH-6 (Val203, 76.7%) are responsible to keep the inhibitor in the binding pocket. In addition, the AH-6partial unfolding, as discussed previously, could be due to the hydrophobic contact of high occupancy between this inhibitor and Val203.

Regarding to TCL and its derivatives (8PC, JPM, JPL, and JPJ), in InhA-NADH-TCL (closed conformation) (2B35, Table 3, Figure 9A), TCL (–OH, H-bond donor) makes an H-bond of high occupancy with NADH (O51, H-bond acceptor, 96.8%) and hydrophobic contact of medium occupancy with Val203 (AH-6, 40.7%). Therefore, the lowest inhibitory potency of TCL could be due to the absence of direct H-bond interaction between inhibitor and enzyme, since this inhibitor interacts by H-bond only with the cofactor, in addition, it makes hydrophobic contacts with only one residue, which may explain the partial unfolding of AH-6, as discussed before. In InhA-NADH-8PC (open conformation) (3FNE, Table 3, Figure 9B), there are two water-bridges, one comprising 8PC phenol hydroxyl group (PhOH-HOH, 98.5%), Asp148 carboxylate group (HOH-Asp148, 76.2%), and Tyr158 (HOH-Tyr158, 96.2%), and the other comprising 8PC pyridine nitrogen atom (HOH-N1, 57.7%), Ile194 backbone (HOH-Ile194, 38%), and NADH nicotinamide carbonyl group (HOH-NADH, 65.29%). Even though HOH-Asp148 interaction corresponds to a charge-reinforced H-bond, there is no direct H-bond interaction between inhibitor and enzyme amino acids. In addition, there are hydrophobic contacts of high occupancy (92%) comprising Met161 (UAH-5) and both phenol and pyridine rings of 8PC, while the hydrophobic contacts with Ile202 (30.5%) may be contributing to partial unfolding of AH-6.

In InhA-NADH-JPM (closed conformation) (3FNF, Table 2, Figure 9C), JPM (phenol hydroxyl group) makes H-bonds with Tyr158 (42.0%) and with NADH ribose hydroxyl group (O43, 27.2%) close to the nicotinamide ring. Moreover, this inhibitor has the same hydrophobic contacts observed for the most potent compound (TCU), similarly contributing to maintaining the AH-6 secondary structure, as discussed before. In InhA-NADH-JPL (closed conformation) (3FNG, Table 2, Figure 9D), JPL (phenol hydroxyl group) makes H-bonds with Tyr158 (73.6%) and Lys165 (67%), as seen for JPM and 8PS, respectively, and this inhibitor also makes hydrophobic contacts of high (Met103, 94.4%) and low (Ala198, 4.0%) occupancies. The H-bond interactions with Lys165 may favor the hydrophobic contacts with Met103 (MLP-4), reducing the hydrophobic contacts of JPL with Ala198 (AH-6). Finally, as with the previous complex, in InhA-NADH-JPJ (closed conformation) (3FNH, Table 2, Figure 9E), JPJ makes the same H-bond interactions with NADH (25.0%) and Tyr158 (23.7%), but both of low occupancy during the last 5 ns of simulation. On the other hand, JPJ makes hydrophobic contacts of high occupancy with Met103 (100%), probably because phenethyl group increases the conformational freedom, allowing a sandwich-like arrangement, comprising Met103, Tyr158, and the phenethyl group of JPJ.

### 2.4. Steric and Electrostatic Interactions of the Holoenzyme-Inhibitor Complexes

**Figure 10:**
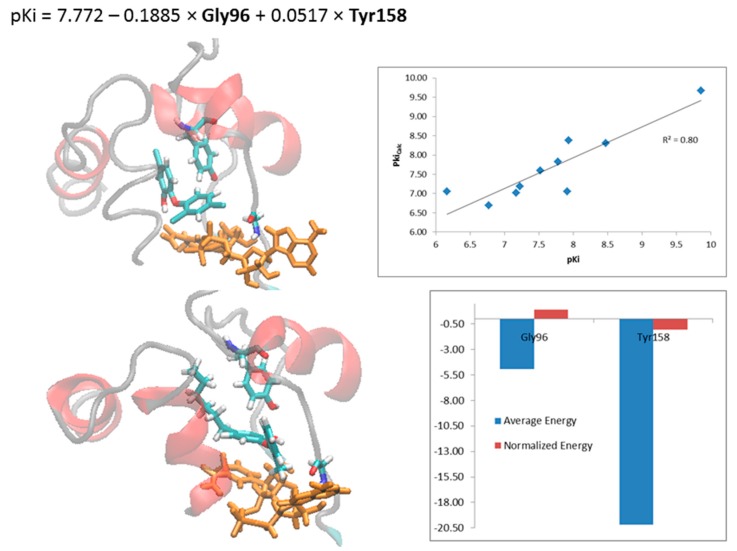
(**A**) Linear equation correlating *pK*_i_ with Gly96 and Tyr158 descriptors (E_C_ + E_LJ_) obtained by PLS/GFA analysis and graphic of the experimental *versus* calculated *pK*_i_. (**B**) Cross correlation matrix of p*K*_i_ and the Gly96C, Phe97LJ, Gly96 (E_C_ + E_LJ_), Phe97 (E_C_ + E_LJ_) and Tyr158 (E_C_ + E_LJ_) descriptors. Close view of the active site of holoenzyme (InhA-NADH) in complex with (**C**) TCU and (**D**) TCL, showing only residues (Gly96 and Try158, yellow; Phe97, gray) with *R* ≥ |0.60| to p*K*_i_ values.

The correlation of the biological response (p*K*_i_) with the steric (E_LJ_), electrostatic (E_C_) and total (E_LJ_ + E_C_) interaction energies from the MD simulations was done by the PLS/GFA analysis using the WOLF program [51]. In the best linear equation (p*K*_i_ = 7.772 − 0.1885 × **Gly96** + 0.0517 × **Tyr158**; *R*^2^ = 0.80; *n* = 10; Figure 10) the selected descriptors, Gly96 and Tyr158, correspond to the total energy (E_LJ_ + E_C_). Descriptor contribution to the activity (p*K*_i_) should be analyzed by considering the energy value and its coefficient signals (positive and negative). As the Gly96 descriptor has negative signals of value and coefficient, it increases the p*K*_i_, *i.e.*, this descriptor contributes increasing the potency. However, Tyr158 descriptor decreases the potency, since it has negative value and positive coefficient.

Simple correlation analysis between p*K*_i_ and independent variables (Supplemental Material) shows high correlation (*R* ≥ |0.80|) between Gly96 descriptor and activity (*R* = −0.85) (Figure 10A), mainly due to electrostatic term (Gly96C, *R* = −0.82), since there is not significant correlation between p*K*_i_ and steric term (Gly96LJ, *R* = −0.38). According to Table 3, only TCU interacts with Gly96 for 26.5% of the time by H-bond, thus, other types of electrostatic interaction are more important in explaining the high correlation. In the case of Tyr158 descriptor (Figure 10B), the correlation with p*K*_i_ is not as high (*R* = 0.64) and is not related separately to any of these terms: steric (Tyr158LJ, *R* = 0.20) and electrostatic (Tyr158C, *R* = 0.39). According to Table 3, Tyr158 makes both types of interactions: H-bond (e.g., 73.6% of the time with JPL) and hydrophobic (e.g., 71.1% of the time with JUS). Despite not being selected in the best equation, Phe97 descriptor also shows some correlation with activity (*R* = −0.60), mainly due to steric contribution (Phe97LJ, *R* = −0.65), but not to electrostatic term (Phe97C, *R* = −0.24).

In the cross correlation matrix of the descriptors (Figure 10B), it is possible to identify pairs of descriptors that may be contributing redundantly in the explanation of the biological response. This matrix has been calculated only for the descriptors that have *R* ≥ |0.60| with activity, *i.e.*, Gly96 (E_C_ and E_LJ_ + E_C_), Phe97 (E_LJ_ and E_LJ_ + E_C_) and Tyr158 (E_LJ_ + E_C_). The best equation descriptors (Gly96 and Tyr158) are poorly correlated (*R* = −0.48), indicating that each descriptor provides almost unique information to the model. The Gly96/Phe97 (*R* = 0.66) and Gly96/Phe97LJ (*R* = 0.65) descriptors are not highly correlated, but have some correlation, which can be explained by the spatial proximity of these residues. On the other hand, highly correlated descriptors, such as Phe97/Phe97LJ (*R* = 0.97) and Gly96/Gly96C (*R* = 0.93), correspond to the same amino acid.

Figure 10 shows the holoenzyme in complex with the most (Figure 10C) and least (Figure 10D) active compounds, highlighting only the amino acids with *R* ≥ |0.60| with p*K*_i_. The proximity of TCU with Gly96 (Figure 10C) causes conformational change of the cofactor adenine group, favoring a pi-stacking interaction between Phe97 and the purine ring, while for TCL (Figure 10D), this conformational change is not observed.

### 2.5. Final Considerations

The analysis of the intermolecular interactions of the holoenzyme-inhibitor complexes allows us to establish a qualitative relationship between the maintenance of secondary structures that compose the substrate-binding pocket, particularly AH-6 (α-helix-6), and the relative potency of inhibitors. In general, the most potent compounds (high p*K*_i_ values) interact through H-bonds directly with Gly96 backbone (TCU, p*K*_i_ = 9.86 M) or the lateral chain of Lys165 (8PS, p*K*_i_ = 8.47 M) or Tyr158 (JPM, p*K*_i_ = 7.93 M and 5PP, p*K*_i_ = 7.91 M. On the other hand, the less potent compounds (low p*K*_i_ values) interact by H-bond only with NADH cofactor (TCL, p*K*_i_ = 6.16 M) or they also interact with enzyme amino acids, but only through water-bridge H-bonds (JUS, p*K*_i_ = 6.77 M). This behavior is in agreement with literature, where TCL binds weakly to holoenzyme when the cofactor is reduced, since no significant interaction was identified [30,31,40].

Regarding to the enzyme conformational state, in the holoenzyme-inhibitor complex, the open conformation is favored when the inhibitor makes hydrophobic contacts of high occupancy with residues from AH-5 (Tyr158 or Met161). In addition, water-bridge H-bonds only occurs when the mobility of LP-6 and AH-7 intercession increases, which favors an open conformation, consequently, there is a decrease in the biological response. Furthermore, when inhibitor makes hydrophobic contacts with only one residue from AH-6 (Ala198, Ile202, or Val203) with occupancy of 30% or more, it induces the unfolding of this secondary structure, while hydrophobic contacts with two residues or more favor the maintenance of the AH-6 secondary structure. Overall, the most potent compounds are those in which the enzyme assumes a closed conformational state, similar to holoenzyme, as is the case of the four most potent inhibitors: TCU (p*K*_i_ = 9.86 M), 8PS (p*K*_i_ = 8.47 M), JPM (p*K*_i_ = 7.93 M), and 5PP (p*K*_i_ = 7.91 M). On the other hand, the less potent compounds are usually those in which the enzyme has an open conformational state (somewhat similar to that adopted in the enzyme-cofactor-substrate complex), as is the case of the two less potent inhibitors: TCL (p*K*_i_ = 6.16 M) and JUS (p*K*_i_ = 6.77 M).

Regarding the steric (E_LJ_) and electrostatic (E_C_) interactions between inhibitors and residues, according to the linear equation shown in Figure 10 (p*K*_i_ = 7.772 − 0.1885 × **Gly96** + 0.0517 × **Tyr158**; *R*^2^ = 0.80; *n* = 10), the total energies (E_LJ_ + E_C_) related to Gly96 and Tyr158 are able to explain 80% of the biological response variance. Interactions with Gly96, especially electrostatic, contribute to increasing the p*K*_i_ value, while both types of interactions with Tyr158 decrease the p*K*_i_ value.

## 3. Experimental Section

### 3.1. Structure of Protein Complexes Selection

The X-ray crystal structures of InhA in complex with cofactor (NADH/NAD+), a substrate mimic (THT), and 10 inhibitors (diphenyl ethers derivatives) were retrieved from the Protein Data Bank (PDB), using the following PDB IDs (enzyme-cofactor, enzyme-cofactor-substrate, and enzyme-cofactor-inhibitor; Resolution, R, Å): 2AQ8 (InhA-NADH; *R* = 1.92 Å) [26]; 1BVR (InhA-NAD-THT; *R* = 2.80 Å) [27]; 2X23 (InhA-NAD-TCU; *R* = 1.81 Å) [25]; 2B35 (InhA-NAD-TCL; *R* = 2.30Å), 2B36 (InhA-NAD-5PP; *R* = 2.80 Å), 2B37 (InhA-NAD-8PS; *R* = 2.60Å) [23]; 3FNF (InhA-NAD-JPM; *R* = 2.30Å), 3FNG (InhA-NAD-JPL; *R* = 1.97 Å), 3FNE (InhA-NAD-8PC; *R* = 1.98 Å), 3FNH (InhA-NAD-JPJ; *R* = 2.80 Å) [24]; 4OIM (InhA-NAD-JUS; *R* = 1.85Å) [16]; and 4OXY (InhA-NAD-1TN; *R* = 2.35Å) [28]. As the PDB files 2B35 and 2B37 had missing atomic coordinates of amino acid residues located in the active site, 2X23 was used for the molecular docking simulations of the corresponding TCL and 8PS inhibitors.

The 3D structures of the ligands (cofactor, substrate, and inhibitors) were extracted from their respective complexes with InhA according to the following PDB ligand codes: NAI (NADH, reduced cofactor form, 1,4-dihydronicotinamide-adenine-dinucleotide), THT (a substrate mimic, *trans*-2-hexadecenoyl-(*N*-acetyl-cysteamine)-thioester), TCU (5-hexyl-2-(2-methylphenoxy)phenol), TCL (triclosan, 5-chloro-2-(2,4-dichlorophenoxy)phenol), 5PP (5-pentyl-2-phenoxy-phenol), 8PS (5-octyl-2-phenoxy-phenol), JPM (5-benzyl-2-(2,4-dichlorophenoxy)phenol), JPL (5-(cyclohexylmethyl)-2-(2,4-dichlorophenoxy)phenol), 8PC (2-(2,4-dichlorophenoxy)-5-(pyridin-2-ylmethyl)phenol), JPJ (2-(2,4-dichlorophenoxy)-5-(2-phenylethyl)phenol), JUS (2-(2-cyanophenoxy)-5-hexylphenol), and 1TN (5-hexyl-2-(2-nitrophenoxy)phenol) (Figure 1).

In all calculations, it was used the cofactor in its reduced form (NADH). The 3D structures of two diphenyl ethers inhibitors (1TN and JPL) have been corrected due to disagreements with experimental data published in the original articles [16,24]. The n-pentyl group of 1TN was corrected as n-hexyl group, while the 1,5-dien-1-ylmethyl group of JPL was corrected as cyclohexylmethyl group.

### 3.2. Biological Data

The biological activity (measured as the half-maximal inhibitory concentration, IC_50_) of the diphenyl ethers derivatives was compiled from various papers and it was determined at different concentrations of the enzyme (InhA), cofactor (NADH), and C12 fatty acyl substrate (2-*trans*-docenoyl-coenzyme A, DD-CoA) [16,23,24,25,28]. This concentration variation, mainly of enzyme, may lead to misinterpretations of the actual inhibitor potency, as in case of TCU having two IC_50_ values (5.3 and 50.3 nM) depending on the InhA concentration (10 and 100 nM, respectively) [25]. Therefore, to avoid comparisons of IC_50_ values measured at relatively different experimental conditions, these data were normalized by their conversion to the inhibition constant (*K*_i_) according to Equation 1 [29]. This is because the diphenyl ethers derivatives are classified as uncompetitive inhibitors that binds slowly in the same cavity of the substrate [50].

(1) $$Ki=\frac{\left(IC_{50}-E/2\right)}{\left(Km/S+1\right)}$$

In Eqation 1, *K*_i_ is the inhibition constant; IC_50_ is the half-maximal inhibitory concentration; *E* and *S* are the concentrations of enzyme (E) and substrate (S), respectively; and *K*_m_ is the Michaelis-Menten constant for each E-S system. In the InhA inhibition assays using DD-CoA as substrate and NADH as cofactor, *K*_m_ is equal to 29 µM [23]; and the InhA (E) and DD-CoA (S) concentration values varied between 1–100 µM and 25–300 µM, respectively [16,23,24,25,52]. Finally, to allow a linear and direct comparison of the biological activity of these derivatives, the *K*_i_ value was converted to its corresponding negative logarithm (p*K*_i_ = −Log*K*_i_) expressed in Molar (M) unit (Table 1).

### 3.3. Molecular Docking Simulation

Before the molecular dynamics (MD) simulations, molecular docking was performed for TCL, 8PS, 1TN, and JPL in order to obtain a starting atomic coordinate of them with holoenzyme (InhA-NADH). Firstly, the 3D structures of these compounds were submitted to the default conformational analysis process of the Spartan program (version 10, Wavefunction, Inc., Irvane, CA, USA) [53], using the RM1 semi-empirical method [54]. The most stable conformation of each compound was submitted to a single-point calculation by the DFT/B3LYP method, using the 6-31+G(d) base function available on Spartan, in order to derive the partial atomic charges.

These compounds were docked within the InhA-NADH complex (PDB ID: 2X23), excluding TCU inhibitor and all water molecules, using the Molegro Virtual Docker (MVD) program [55], according to the previously works published by our group [56,57]. The MVD automatic preparation module was used to correct the atoms types and bond orders, to add the hydrogen atoms and to assign the default atomic partial charges in the protein structure.

Potential binding sites (cavities) were detected using the grid-based cavity prediction algorithm. The population size, maximum interactions, scaling factor, and crossover rate were set to 150, 1500, 0.50, and 0.90, respectively. For each complex, we performed 100 independent runs with the MolDock optimizer algorithm, returning five solutions (poses, *i.e.*, conformation and orientation) for each run. The MolDock score function with a grid resolution of 0.30 Å was used to precompute score grids for rapid dock evaluation. Guided differential evolution and a force-field-based docking scoring function were used to search for the binding orientation and conformation of each candidate molecule. The best pose of each inhibitor was selected for the subsequent MD simulations.

To guarantee the reliability of the docking protocol previously described, the TCU inhibitor was removed and docked back into the active site of InhA-NADH. The root-mean-square deviation (RMSD) calculated between the best pose and the original coordinates was less than 2 Å, an acceptable value as suggested by different authors in literature [58,59,60,61,62].

### 3.4. Molecular Dynamics Simulations

Molecular dynamics (MD) simulations of twelve aqueous systems of the enoyl-acyl carrier protein (ACP) reductase enzyme from *Mycobacterium tuberculosis* (InhA) bound to the NADH cofactor were carried out using the GROMACS 4.5 package [63,64,65] with OPLS/AA forcefield [66]. It was investigated one binary (holoenzyme) and eleven ternary (holoenzyme-ligand) complexes. The ligands are a substrate mimic (*trans*-2-hexadecenoyl-(*N*-acetyl-cysteamine)-thioester, THT) and ten diphenyl ethers derivatives, including triclosan (5-chloro-2-(2,4-dichlorophenoxy)phenol, TCL), which are potent InhA inhibitors.

The ligands (cofactor, substrate, and inhibitors) topology were created using the AnteChamber PYthon Parcer InterfacE (ACPYPE) tool [67], and the ACPYPE missing parameters were generated by the MKTOP software [68]. The partial atomic charges were assigned according to the AM1-BCC parameters [69], through the ANTECHAMBER package [70,71]. The holoenzyme and holoenzyme-ligand complexes were inserted and centered into the cubic periodic box of spc216 water molecules and it was considered the SPC/E water model [72], thus each complex was neutralized by six Na^+^ counter-ions. Each complex was submitted to a preliminary optimization using the steepest descent algorithm with position restrained (PR) of the ligands (cofactor, substrate, and inhibitors) and convergence criteria of 1000.00 kJ·mol^−1^·nm^−1^, followed by steepest descent without PR, and conjugate gradients until an energy of 100.00 kJ·mol^−1^·nm^−1^. Then, the minimized complexes were submitted to 1000 ps of MD, at 300 K, considering NVT and NPT state with PR to the entire system, except to water molecules, using the V-rescale thermostat [73], and the Parrinello-Rahman scheme [74] for pressure coupling. The PR was applied to guarantee the distribution of the solvent molecules around the protein. All bonds involving hydrogen atoms in the complexes were frozen by the LINCS scheme [75], the long-range electrostatic interactions were treated using the PME algorithm (Particle-Mesh Ewald) [76,77], and a cut-off value of 1 nm was applied for the van der Waals and Coulomb interactions. Afterward, it was carried out 20 to 40 ns of MD simulations without any restriction, using 2 fs of integration time and a cut-off of 10Å for long-distance interactions.

The criteria used to finalize the MD simulations followed a rigorous protocol to determine if the system is indeed in an equilibrium state. Thus, from 10 ns, we calculated the RMSD value during every 5 ns (e.g., from 10 to 15 ns, from 15 to 20 ns, *etc.*), verifying if the RMSD variation was lower than 1.5 and 1.0 Å for the protein backbone and ligand, respectively. In case of InhA-NADH-THT, we establish the RMSD variation should be less than 2 Å, due to the high degree conformational freedom of the substrate mimic.

### 3.5. Molecular Dynamics Analysis

The root mean standard deviation (RMSD) calculation, cluster analysis, mean structure extraction, interatomic distance measurements, and hydrogen bond analysis were performed using diverse modules available in GROMACS 4.5 [63,64,65]. The H-bonds (D-H…A) were computed considering the cutoff distance between donor (D) and acceptor (A) atoms until 0.35nm and the cutoff H-D-A angle until 30°. The H-bonds frequency was calculated using hbmap2grace package [78]. The cutoff value for distance (nm) between non-hydrogen atoms of ligand, cofactor and residues was until 0.40nm. The occupancy percentage (%) for H-bonds and interatomic distances were calculated only for the last 5 ns of simulation. The 3D structure was rendering using VMD program [79]. The Grace program was used to plot the RMSD graphics [80]. The 3D root mean square fluctuation (RMSF) or sausage representation was created for the last 5 ns, using the MOLMOL package [81].

### 3.6. Steric and Electrostatic Interactions Calculations of the Holoenzyme-Inhibitor Complexes

The steric (Lennard-Jones, LJ) and electrostatic (Coulomb, C) interaction energies (E_LJ_ and E_C_) between the inhibitors and holoenzyme were calculated only for the binding site residues, using the average energy of the 100 conformations of the most populated cluster from the last 5 ns of each simulation. In order to estimate the influence of these descriptors (independent variables) on the biological response (p*K*_i_) (dependent variable), we considered the steric (E_LJ_), electrostatic (E_C_) and total (E_LJ_ + E_C_) average energies by residue for each inhibitor. The 192 descriptors resulting were submitted to a combined Partial Least Squares (PLS) and Genetic Function Approximation (GFA) analysis available in the WOLF program [51]. In the PLS/GFA calculations, it was considered three principal components, population of 600–1000 equations, crossover operations of 50,000–500,000, mutation rate of 100%, and smoothing factor of 0.1–1.0.

The variance (σ^2^) of the p*K*_i_ values shows a small dispersion (σ^2^ = 1.02), which means that descriptors with small variance do not contribute with relevant information to the energy-activity relationship. Thus, we discarded descriptors with σ^2^ < 0.30. Then, we submitted the 71 descriptors resulting to the WOLF program, in order to generate equations that may explain the biological response. Due to the limited number of compounds (*n* = 10), we selected only equations containing two descriptors. In order to select descriptors correlated with p*K*_i_ and intercorrelated, we calculated the simple correlation (R) by the cross-correlation matrix of the descriptors and p*K*_i_ (Supplementary Material).

## 4. Conclusions

The MD simulations showed the importance of the H-bond and hydrophobic contact interactions between holoenzyme and ligands, responsible for structural changes and the steric and electrostatic interaction energies to identify important residues correlated with the biological response of diaryl ethers derivatives. Analysis of 2AQ8 and 1BVR revealed that the open and closed conformational states of InhA are related to the AH-6 motion, while the solvent-inhibitor interaction is related to the mobility of LP-6/AH-7 intersection that works as a lid. The enzyme assumes an open conformation mainly when the inhibitor makes: (i) H-bonds with the cofactor, (ii) indirect H-bonds (water-bridge H-bonds) with enzyme residues as a consequence of the lid opening, and (iii) hydrophobic contacts (>30% of occupancy) with AH-5. All these interactions are related to a decrease in the inhibitor affinity by the holoenzyme. Moreover, the protein-inhibitor total steric (E_LJ_) and electrostatic (E_C_) interaction energies, related to Gly96 and Tyr158, are able to explain 80% of the biological response variance according to the best linear equation, p*K*_i_ = 7.772 − 0.1885 × **Gly96** + 0.0517 × **Tyr158** (*R*^2^ = 0.80; *n* = 10), where interactions with Gly96, mainly electrostatic, increase the biological response, while those with Tyr158 decrease. Taken together, our results demonstrate the importance in studying the enzyme-cofactor-inhibitor dynamic behavior, considering the structural changes, solvent influence, and interaction energies for understanding the biological activity. These results will help to understand the structure-activity relationships and to design new and more potent anti-TB drugs.

## Supplementary Materials

Supplementary materials can be found at http://www.mdpi.com/1422-0067/16/10/23695/s1.
